# High‐throughput mutational analysis of F_1_ ‐ATPase by integrated cell‐free protein synthesis and single‐molecule rotation assay

**DOI:** 10.1002/pro.70699

**Published:** 2026-07-10

**Authors:** Mai Taguchi, Tatsuya Oya, Hiroshi Ueno, Hiroyuki Noji

**Affiliations:** ^1^ Department of Applied Chemistry, Graduate School of Engineering The University of Tokyo Tokyo Japan; ^2^ Research Institute of Planetary Health (RIPH) The University of Tokyo Tokyo Japan

**Keywords:** ATP synthase, cell‐free protein synthesis, high‐throughput screening, mechanochemical coupling, protein engineering, rotary molecular motor, saturation mutagenesis, single‐molecule analysis

## Abstract

F_1_‐ATPase (F_1_) is a rotary molecular motor that hydrolyzes adenosine triphosphate (ATP) to drive rotation of the central subunit against the surrounding stator ring. Single‐molecule rotation assays of mutated F_1_s have elucidated the roles of residues and regions in the chemo‐mechanical coupling mechanism, yet comprehensive mutational dissection has been constrained by low‐throughput workflows. Here we established a high‐throughput platform that integrates cell‐free protein synthesis with a multiplexed single‐molecule rotation assay. We applied the method to saturation mutagenesis of βE190, the catalytic general base, and βY307, a highly conserved residue at the entrance of a putative phosphate‐release tunnel. βE190 substitutions yielded sharply defined outcomes. Only aspartate retained partial activity, highlighting a decisive requirement for negative charge at this catalytic position. By contrast, βY307 variants showed broader tolerance, with activity correlating with side‐chain size and hydrophobicity. Moreover, the inactive‐state fraction increased for smaller substitutions at βY307, suggesting that the putative tunnel is not catalytically relevant for turnover but may instead be related to occasional phosphate‐release events associated with ADP inhibition. Notably, this workflow completes the entire cycle from protein synthesis to functional analysis within 10 h, enabling rapid, comprehensive mutational profiling of rotary ATPases.

## 1 INTRODUCTION

F_o_F_1_‐ATP synthase is a ubiquitous molecular machine responsible for ATP production in oxidative phosphorylation and photosynthesis (Boyer, 1997; Kuhlbrandt, 2019; Noji et al., 2017; Walker, 2013). It harnesses proton motive force across biological membranes to drive the synthesis of ATP from ADP and inorganic phosphate. Structurally, the enzyme consists of two coupled rotary motors: the membrane‐embedded F_o_, in which the rotor ring rotates upon proton translocation, and the soluble F_1_, which couples the catalytic reaction to rotation of the central stalk. Rotation of the central stalk transmits torque between F_o_ and F_1_, ensuring tight chemo‐mechanical coupling (Macdonald & Ashby, 2025; Noji et al., 2017).

When isolated from F_o_ or operating under low proton motive force, the F_1_ portion hydrolyzes ATP instead of synthesizing it; therefore, termed F_1_‐ATPase and hereafter referred to as F_1_ for simplicity. The minimal complex of F_1_ as the rotary motor is the α_3_β_3_γ subcomplex composed of the α_3_β_3_ stator ring and the central γ subunit (Abrahams et al., 1994; Noji et al., 1997). Although the catalytic sites are formed at the α–β interfaces, most residues forming these sites are located on the β subunit. The β subunit undergoes large conformational transitions during the catalytic cycle, and these conformational changes are central to torque generation during ATP hydrolysis (Noji & Ueno, 2022).

Rotation of F_1_ hydrolyzing ATP was directly visualized by single‐molecule rotation assays, validating Boyer's binding‐change mechanism (Noji et al., 1997). In the rotation assay, the α_3_β_3_ stator ring is immobilized on a glass surface, and a rotation probe such as an actin filament, microbead, or gold nanoparticle is attached to the protruding part of the γ subunit (Noji et al., 2017). The single‐molecule rotation assay has made significant contributions to elucidating the rotary catalysis mechanism of F_1_, resolving steps and substeps and identifying catalytic events coupled with individual substeps (Bilyard et al., 2013; Frasch et al., 2022; Kobayashi et al., 2020; Noji et al., 2017; Suzuki et al., 2014; Zarco‐Zavala et al., 2020). These studies established the basic chemo‐mechanical coupling scheme of F_1_ and revealed diversifications of the scheme among species. The rotation assay also revealed that F_1_ makes a slow transition between an actively rotating state and an inhibitory state pausing rotation, called adenosine diphosphate (ADP) inhibition (Hirono‐Hara et al., 2001; Lapashina & Feniouk, 2018).

Mutagenesis studies using the rotation assay revealed the roles of catalytic residues, such as the general base (βE190 in numbering of F_1_ from thermophilic *Bacillus* PS3, TF_1_), lysine in Walker motif A, and the arginine finger (αR364) (Komoriya et al., 2012; Shimabukuro et al., 2003; Watanabe et al., 2014). Some structurally distinct regions were also investigated to elucidate how torque is transmitted at the rotor–stator interface (Furuike et al., 2008; Tanigawara et al., 2012; Usukura et al., 2012). However, conventional approaches relying on bacterial expression and protein purification are labor‐intensive and low‐throughput. As a result, typically only a handful of variants have been examined at the single‐molecule level for each residue, limiting our ability to determine how physicochemical properties such as charge, polarity, or steric bulk shape F_1_ catalysis. To overcome these limitations, exploiting the rapid and simple nature of a cell‐free transcription and translation (TXTL) system (Shimizu et al., 2001). In conventional workflows, bacterial expression and purification of each mutant requires days per construct. By contrast, cell‐free TXTL systems enable direct and rapid protein synthesis in a tube, eliminating cell culture, harvest, and lysis processes. In particular, the PURE system, composed of purified components, allows rapid preparation of many variants in parallel (Shimizu et al., 2001); because the PURE components can be easily removed by washing, rapid preparation and assay are possible. We conceived that integrating cell‐free TXTL with the PURE system and the rotation assay would greatly improve the throughput of mutant analysis. Therefore, as a first step, we developed a cell‐free TXTL protocol for preparing functional F_1_ proteins applicable to single‐molecule rotation assays. We then combined this protocol with a multiplexed single‐molecule rotation assay to establish a high‐throughput platform for systematic functional profiling of F_1_ variants. This framework allowed us to compare the full substitution set at a given residue and thereby identify both global trends and informative exceptions. To validate the performance of the platform, we performed saturation mutagenesis of βE190, a residue whose catalytic role has already been partially characterized. We next applied the platform to βY307, a highly conserved residue recently suggested by structural analyses to be catalytically important (Motohashi et al., 2025) and positioned near a putative phosphate‐release tunnel (Sobti et al., 2021). Previous studies examined limited substitutions of βY307 in TF_1_ and equivalent tyrosine residues in *Escherichia coli* and chloroplast F_1_, including their effects on ATPase activity (Chen & Frasch, 2001; Parsonage et al., 1987; Yagi et al., 2009). However, comprehensive saturation mutagenesis of this residue using single‐molecule rotation assays had not been performed. We therefore selected βY307 as a proof‐of‐concept target to examine how the present platform can reveal both catalytic and regulatory effects of mutations.

## 2 RESULTS

### 2.1 Cell‐free expression for rotation assay

**FIGURE 1:**
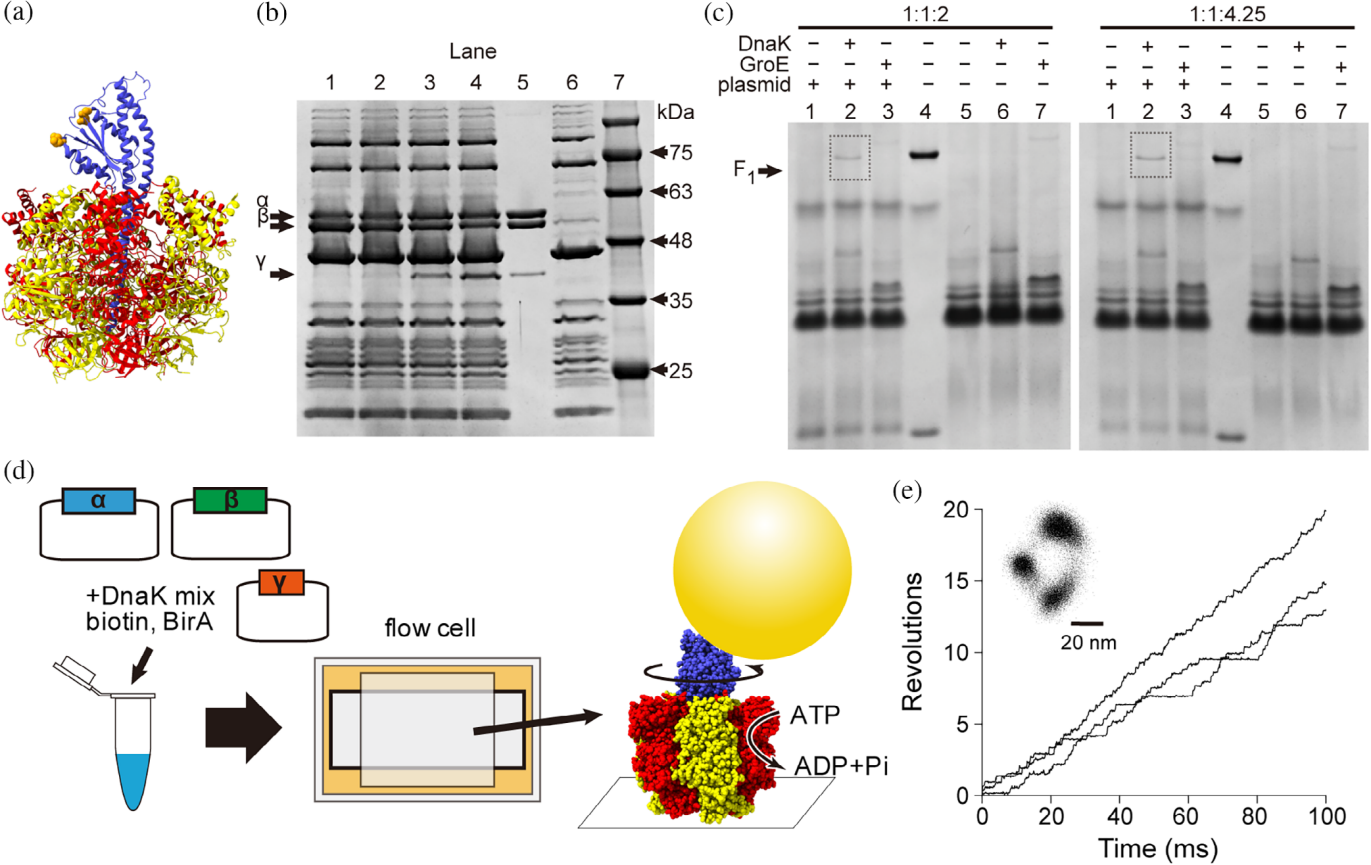
Cell‐free expression of F_1_‐ATPase. (a) Avi‐tag insertion sites in the F_1_ γ subunit. Avi‐tag sequences were inserted between residues A108 and S109, and between G163 and T164 (yellow). (b) SDS‐PAGE of cell‐free expression products. Lanes 1–4: Reactions with expression vectors for the α, β, and γ subunits mixed at DNA molar ratios of 1:1:1, 1:1:2, 1:1:4.25, and 1:1:8, respectively; Lane 5: Purified F_1_; Lane 6: PURE system only; Lane 7: molecular‐weight marker. (c) Native PAGE of cell‐free expression products with or without chaperone systems. Reactions containing the α, β, and γ expression plasmids at an α:β:γ DNA molar ratio of 1:1:2 (left) and 1:1:4.25 (right) were analyzed. In each panel, Lane 1, plasmids only (no added chaperone); Lane 2, plasmids + DnaK Mix; Lane 3, plasmids + GroE Mix. Lane 4, purified F_1_; Lane 5, PURE system only (no plasmids, no added chaperone); Lane 6, PURE system only + DnaK Mix; Lane 7, PURE system only + GroE Mix. (d) Schematic of cell‐free single‐molecule rotation assay. (e) Rotation trajectories of cell‐free synthesized F_1_ at 2 mM ATP. (*Inset*) representative *x*–*y* plot of the centroid of the rotating probe.

For cell‐free expression of the F_1_ complex for rotation assay using the PURE system, we constructed three separate plasmids encoding the α, β, and γ subunits so that the expression yield of each subunit could be independently adjusted. An Avi‐tag sequence, a 15‐amino acid peptide specifically recognized by the *E. coli* biotin ligase BirA (Beckett et al., 1999), was genetically introduced into the γ subunit for specific enzymatic biotinylation. Two Avi‐tag sequences were inserted: one between A108 and S109 and the other between G163 and T164, following our previous two‐point probe‐coupling design used in chemically biotinylated rotation assays, to support stable and efficient torque transmission from the γ subunit to a nanoparticle used as the rotation probe (Figure 1a; see also Figure S1 for an AlphaFold model of the Avi‐tagged γ subunit). We examined the DNA mixing ratio for cell‐free expression using α:β:γ ratios of 1:1:1, 1:1:2, 1:1:4.25, and 1:1:8. Comparison with purified F_1_ on sodium dodecyl sulfate–polyacrylamide gel electrophoresis (SDS‐PAGE) indicated that the 1:1:2 and 1:1:4.25 conditions most closely approximated the overall band balance, although the relative β‐band intensity showed some variation between independent in vitro transcription and translation (IVTT) reactions (Figures 1b and S2a). The 1:1:4.25 condition tended to produce a slightly stronger γ band than purified F_1_, whereas the 1:1:2 condition showed a slightly lower γ level. We therefore selected 1:1:2 and 1:1:4.25 as candidate conditions for further evaluation of F_1_ complex formation by native PAGE. However, under these conditions, complex formation remained undetectable. We then tested commercially available molecular chaperone add‐on systems for the PURE system, namely the GroE system (GroEL/GroES) and the DnaK system (DnaK/DnaJ/GrpE) (Niwa et al., 2012), and found that addition of the DnaK system clearly enabled complex formation under both conditions (Figures 1c and S2b). As a reference, in the case of cell‐free expression of F_1_ without the Avi‐tag, the complex was observed even without the DnaK system (Figure S3). This suggests that insertion of the Avi‐tag may have adversely affected folding and/or assembly of the γ subunit, thereby making assistance from the DnaK system necessary. The selective effect of DnaK over GroE is consistent with the possibility that the main bottleneck lies in early folding intermediates or aggregation‐prone states of the expressed γ subunit, which can be suppressed by the DnaK system to maintain an assembly‐competent form.

After expression in the cell‐free TXTL system, the F_1_ complex was treated with BirA for enzymatic biotinylation of the lysine in the Avi‐tag. Western blotting confirmed the specific biotinylation of the γ subunit in a representative reaction (Figure S4). For all subsequent single‐molecule assays, including the multiplexed rotation assay, cell‐free reactions were prepared at an α:β:γ DNA molar ratio of 1:1:2. For the rotation assay, the diluted reaction mixture was introduced into a nickel–nitrilotriacetic acid (Ni‐NTA)‐coated flow chamber, where F_1_ was immobilized via His‐tags on the α_3_β_3_ ring. Components of the PURE system do not carry these His‐tags and were removed during the subsequent washing step. Streptavidin‐coated gold nanoparticles were then introduced into the chamber after washing, followed by ATP infusion to initiate rotation measurements (Figure 1d). For comparison, F_1_ with the Avi‐tag was purified from *E. coli* cells and tested in the rotation assay. Rotation was recorded with a laser dark‐field microscopy system at 10,000 frames per second (fps) with a streptavidin‐coated gold nanoparticle (40 nm in diameter) as a rotation probe (Ueno et al., 2010). The rotation rates were comparable between the cell‐free‐derived and *E. coli*‐derived F_1_ (Table 1). In addition, stepping rotation was also observed for the cell‐free derived F_1_ (Figure 1e). Thus, the functional integrity of cell‐free synthesized F_1_ was confirmed.

**TABLE 1:** Rotation rates of cell‐free expressed F_1_ and *Escherichia coli*‐expressed F_1_.

Protein	Rotation rate (rps)	*N* (molecules)
Cell‐free expressed F_1_	176 ± 42	63
*E. coli*‐expressed F_1_	162 ± 24	58

*Note*: Values are mean ± SD. Cell‐free expressed F_1_ data were combined from two independent PURE synthesis experiments measured over four assays. The *E. coli*‐expressed F_1_ control was measured over two assays.

### 2.2 Multiplexed single‐molecule rotation assay

**FIGURE 2:**
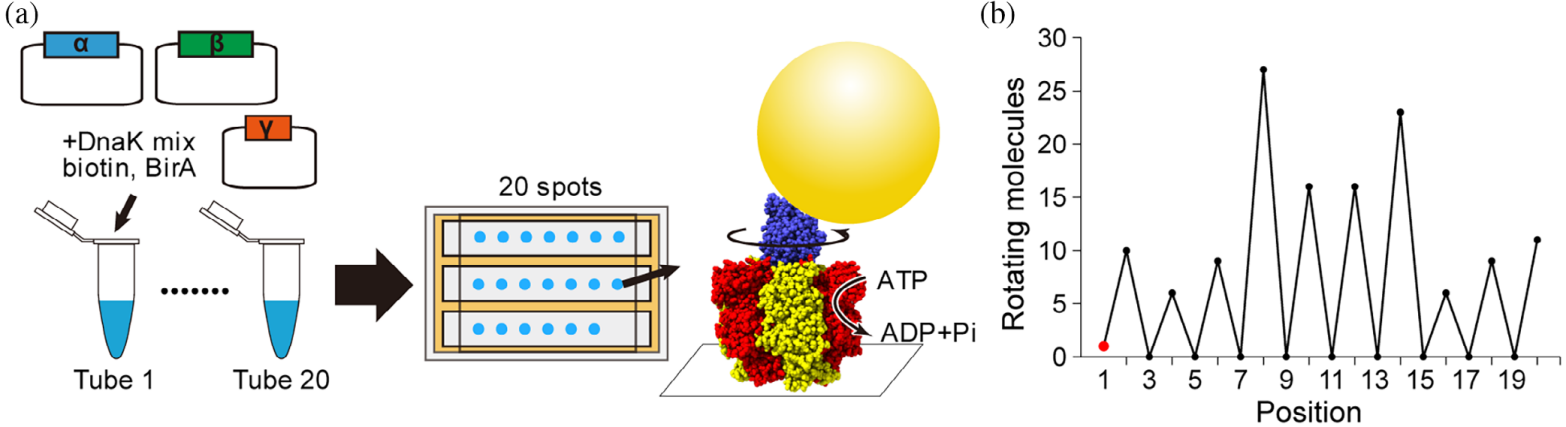
Multiplexed single‐molecule rotation assay and crosstalk evaluation. (a) Schematic of multiplexed single‐molecule rotation assay with multiple spotted PURE reactions. (b) Representative result of the crosstalk evaluation experiment with F_1_(βE190Q) spots (positions 1, 3, 5, 7, 9, 11, 13, 15, 17, and 19) and F_1_(βT165S/G181A) spots (positions 2, 4, 6, 8, 10, 12, 14, 16, 18, and 20). Red marks indicate rare rotation events detected at F1(βE190Q) spots.

To increase throughput, we aimed to establish a multiplexed single‐molecule rotation assay using a flow cell format in which multiple F_1_ variants are immobilized at distinct positions and assayed simultaneously (Figure 2a). For this purpose, 1 μL of tenfold diluted PURE reaction mixtures expressing F_1_ proteins were spotted onto defined areas (approximately 3 to 4 mm in diameter) of a Ni‐NTA‐coated glass coverslip and sealed with a cover glass to form a flow chamber. After a 7‐min immobilization step, the chamber was washed with buffer to remove unbound proteins and residual PURE components lacking His‐tags. Gold nanoparticle probes were then introduced and incubated in the chamber, and rotation measurements were started by subsequent infusion of 2 mM magnesium–ATP complex (Mg‐ATP). Rotational motions of nanoparticles were recorded with laser dark‐field microscopy at 1,000 fps recording rates.

In this format, multiple spots shared a common flow channel, allowing simultaneous solution exchange. Therefore, there was a potential concern of crosstalk caused by F_1_ molecules dissociating from one spot and reaching another. To evaluate this, we prepared two types of PURE solutions: one expressing a mutant F_1_ with a very high frequency of detectable rotation, F_1_(βT165S/G181A) (Muneyuki et al., 2007; Nishizaka et al., 2004), and another expressing a mutant F_1_ with negligible activity, F_1_(βE190Q) (Watanabe et al., 2014). These two solutions were alternately spotted 10 times each, resulting in a total of 20 spots. The entire set of 20 spots was then enclosed within a common flow channel, and solution exchange was performed to conduct the rotation assay. This entire experimental procedure, from PURE synthesis to the rotation assay, was independently repeated three times. As a result, rotation was observed at all 30 F_1_(βT165S/G181A) spots, with an average of 11 rotating particles per spot, whereas 25 of 30 F_1_(βE190Q) spots showed no rotation (Figures 2b and S5). The remaining five F_1_(βE190Q) spots exhibited only one to two rotating particles of F_1_(βT165S/G181A), consistent with rare low‐level carryover/crosstalk from neighboring spots. These results indicate that crosstalk is rare and low‐level under the present assay conditions. Although not completely absent, it does not materially affect the main conclusions of the subsequent multiplexed mutational analysis.

### 2.3 Saturation mutagenesis of βE190 residue

To examine the applicability of the multiplexed rotation assay to the saturation mutagenesis analysis, we analyzed a comprehensive set of βE190 saturation mutants. This residue is often referred to as the “general base” and is well known as one of the most critical residues for catalysis (Noji et al., 2017; Shimabukuro et al., 2003). Moreover, rotation rates of three substitution variants—E190D, E190A, and E190Q—have been reported in previous studies (Shimabukuro et al., 2003; Watanabe et al., 2014). E190D is often used as a model mutant F_1_ with modestly slowed catalysis, showing rotation at ~1 rps (Shimabukuro et al., 2003), whereas E190A and E190Q were reported to be catalytically lethal, showing extremely slow rotation (tens of seconds per revolution) (Watanabe et al., 2014). In our assay, E190D showed detectable rotation at ~1 rps, while E190A and E190Q did not show rotation within the 20 s observation window, consistent with previous reports (Figure S6). Other mutants did not show rotation activity, exhibiting the decisive role of negative charge at this catalytic residue. These results confirm that our system can reliably detect rotational activity above ~1 rps and that its detection threshold is mainly defined by the recording length and frame rate. Importantly, the entire saturation mutagenesis experiment of βE190 was completed within 10 h, including ~3 h for synthesis and ~7 h for single‐molecule assays.

### 2.4 Saturation mutagenesis of βY307 residue

**FIGURE 3:**
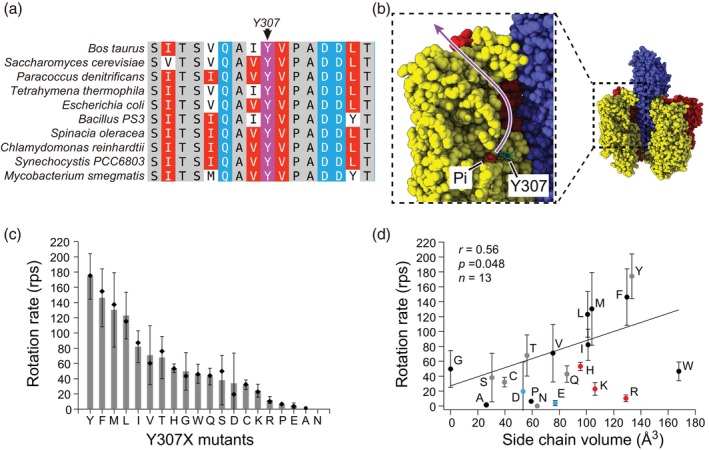
Saturation mutagenesis of βY307. (a) Sequence alignment of the F_1_ β subunits. βY307 is highly conserved across species. (b) Pi‐bound cryo‐EM structure of TF_1_(βE190D) (PDB: 7L1S) showing the putative phosphate‐release tunnel. The α, β, and γ subunits are colored red, yellow, and blue, respectively. One α subunit was omitted for clarity. The inset shows a close‐up view of the proposed alternative Pi path (purple arrow) and the position of βY307 (cyan) near the tunnel entrance. (c) Rotation rates of βY307 site‐saturation mutants measured by a multiplexed rotation assay. All variants carried βT165S/G181A as the background. Data from four independent datasets were combined for analysis. Each dataset was generated from an independent PURE synthesis experiment and subsequent multiplexed rotation assays. Sample sizes (*N*, molecules) were as follows: Y (*N* = 36), F (*N* = 30), M (*N* = 9), L (*N* = 85), I (*N* = 37), V (*N* = 7), T (*N* = 12), H (*N* = 4), G (*N* = 13), W (*N* = 24), Q (*N* = 71), S (*N* = 3), D (*N* = 3), C (*N* = 12), K (*N* = 82), R (*N* = 17), P (*N* = 5), E (*N* = 6), A (*N* = 1). Values are means ± standard deviation (SD), except for βY307A, which is shown as a single point because only a single rotating particle was detected. Black diamonds indicate median values. No detectable rotation was observed for βY307N. (d) Correlation between rotation rate and side‐chain volume for βY307 site‐saturation mutants. Side‐chain volumes were calculated as the residue volume minus the glycine contribution (Harpaz et al., 1994). Black, gray, red, and blue circles represent mutants bearing nonpolar, uncharged polar, positively charged, and negatively charged residues, respectively. βY307N with no detectable rotation was plotted as 0 rps. The line indicates the least‐squares linear fit. Mutants with charged residues were excluded from the fit and from the calculation of Pearson's *r*. βY307A and βY307N were also excluded because only one and no rotating particles were detected, respectively. Pearson's *r*, two‐sided *p*‐value, and *n* (mutants included in the analysis) are shown.

**FIGURE 4:**
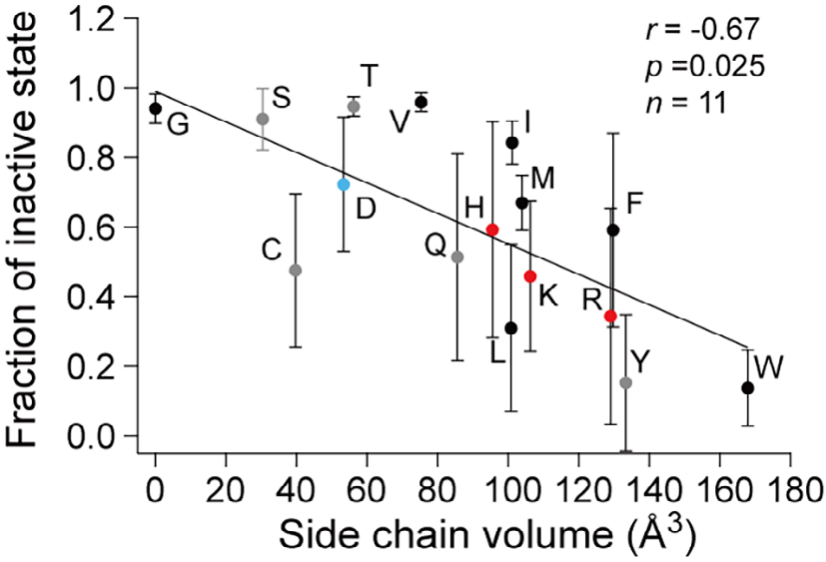
Correlation between inactive‐state fraction and side‐chain volume for βY307 site‐saturation mutants. The inactive‐state fraction for βY307 mutants was plotted against side‐chain volume. Data from four independent datasets were combined for analysis, as in Figure 3c,d. Each dataset was generated from an independent PURE synthesis experiment and subsequent multiplexed rotation assays, and only molecules for which the inactive‐state fraction could be defined were included in the analysis. Black, gray, red, and blue circles represent mutants bearing nonpolar, uncharged polar, positively charged, and negatively charged residues, respectively. Sample sizes (*N*, molecules) were as follows: Y (*N* = 30), F (*N* = 11), M (*N* = 6), L (*N* = 25), I (*N* = 18), V (*N* = 6), T (*N* = 7), H (*N* = 4), G (*N* = 8), W (*N* = 9), Q (*N* = 37), S (*N* = 2), D (*N* = 2), C (*N* = 8), K (*N* = 32), R (*N* = 6). Values are means ± SD, except for βY307S and βY307D, which are shown as the mean with min–max range. Mutants with no detectable rotation or with rotation too slow to define the inactive‐state fraction were not plotted (βY307P, βY307E, βY307A, and βY307N). The line shows the least‐squares linear fit. Mutants with charged residues were excluded from the fit and from the calculation of Pearson's *r*. βY307S, for which only two rotating particles were detected, was also excluded. Pearson's *r*, two‐sided *p*‐value, and *n* (mutants included in the analysis) are shown.

We next focused on βY307, a highly conserved residue located near the entrance of a putative phosphate‐release tunnel identified in the Pi‐bound cryo‐electron microscopy (cryo‐EM) structure of TF_1_ βE190D mutant (Sobti et al., 2021) (Figure 3a,b). In that study, the tunnel was observed as an internal Pi exit path and was proposed as a possible route for Pi release even when the nucleotide‐binding cleft is occupied by ADP. Following that study, we refer to this putative Pi path as the “back door.” Recent molecular dynamics studies further suggested that βY307 can interact with Pi near this region (Motohashi et al., 2025), implying that it functions as the gate of the back door. To test this comprehensively, we carried out saturation mutagenesis of βY307. All βY307 variants were analyzed in the βT165S/G181A background, which is known to attenuate ADP inhibition by reducing entrapment of inhibitory MgADP in a catalytic site (Jault et al., 1996; Masaike et al., 2000). We chose this background to obtain sufficient numbers of actively rotating molecules for high‐throughput comparative profiling. Accordingly, the inactive‐state fractions measured here should be interpreted as residual inactive‐state behavior under ADP‐inhibition‐attenuated conditions, rather than as absolute ADP‐inhibition probabilities of wild‐type‐background F_1_. Nineteen substitutions were introduced and tested with the multiplexed rotation assay (Figure 3c). Unlike βE190, many βY307 variants retained detectable activity, showing active rotation. Bulky hydrophobic substitutions such as Phe and Met retained substantial rotation rates, whereas charged substitutions largely suppressed or abolished activity. Among non‐charged variants for which mean rotation rates could be estimated from multiple rotating particles, rotation rate showed a moderate positive correlation with side‐chain volume (Figure 3d). These results do not support a major role of this putative tunnel in phosphate release during normal catalytic turnover. Instead, an interesting trend was observed when analyzing the inactive‐state fraction. When the inactive‐state fraction was plotted against residue size, a correlation was observed, with smaller substitutions tending to increase the inactive‐state fraction (Figure 4). Considering that ADP inhibition often occurs when phosphate is released prior to ADP, whereas in the canonical scheme phosphate is released after ADP (Watanabe & Noji, 2014), this trend is consistent with the possibility that smaller substitutions at βY307 affect occasional phosphate‐release events linked to inactive‐state behavior associated with ADP inhibition, although the present data do not distinguish whether the primary effect is on entry into, or exit from, the inactive state. Together, these observations indicate that substitutions at βY307 affect both rotational activity and inactive‐state behavior.

## 3 DISCUSSION

In this study, we established a high‐throughput framework for systematic mutational analysis of F_1_‐ATPase by integrating cell‐free protein synthesis with multiplexed single‐molecule rotation assays. This approach reduced the experimental cycle from days to hours and enabled the functional evaluation of dozens of variants within a single run.

Importantly, saturation mutagenesis of both βE190 and βY307 was completed in a single experimental session lasting less than 10 h. Such a one‐shot, comprehensive mutational analysis has not been previously achieved for F_1_ or, more broadly, for any rotary molecular motor. Although a wheat‐germ cell‐free expression and subsequent in vitro motility assay was reported for the kinesin motor (Inoue et al., 2023), comprehensive mutagenesis assays with high‐throughput analysis remain to be performed. The present results indicate that rotations much slower than ~1 rps are essentially undetectable under the present assay conditions, which involve 20‐s observation windows and acquisition at 1,000 fps. However, this detection threshold is not intrinsic to the method: longer observation periods or optimized acquisition settings should allow detection of slower rotations if needed.

From the βE190 saturation mutagenesis, we confirmed its essential catalytic role (Hayashi et al., 2012). Conservative substitution with aspartate (E190D) retained partial activity, though with markedly reduced rotational speed, while other substitutions abolished detectable activity. These results validated the sensitivity of the multiplex assay and demonstrated its reliability in capturing the strong dependence of F_1_ catalysis on this catalytically critical residue.

In contrast, the βY307 saturation mutagenesis revealed a broader mutational profile, extending previous analyses of this conserved residue. Previous mutagenesis studies of βY307 or equivalent tyrosine residues in TF_1_ (Yagi et al., 2009), *E. coli* F1 (Parsonage et al., 1987) and chloroplast F1 (Chen & Frasch, 2001) have examined effects on ATPase activity in bulk assays and the role of this tyrosine as a ligand to the metal of the Mg^2+^–nucleotide complex (Chen & Frasch, 2001). However, these studies did not address the comprehensive mutational landscape of this residue or its effects on single‐molecule rotational behavior. In the present study, saturation mutagenesis of βY307 combined with the multiplexed single‐molecule rotation assay revealed mechanistically informative trends. Overall, bulky hydrophobic substitutions such as phenylalanine and methionine supported rotation, whereas charged substitutions largely suppressed or abolished activity, suggesting that side‐chain volume and hydrophobicity influence catalysis at this site. Notably, however, βY307G retained higher activity than would be expected from a simple monotonic size dependence, suggesting that steric effects alone do not fully explain the behavior at this position. In contrast, smaller side chains increased the inactive‐state fraction during two‐state dynamics between active rotation and inhibitory pauses (Figure 4). In addition, the mean active rotation time between inhibitory pauses showed a moderate positive trend with side‐chain volume among the non‐charged βY307 mutants analyzed in Figure 4 (Table S1; Pearson's *r* = 0.53, two‐sided *p* = 0.095, *n* = 11), although the sample size was limited. Further analysis will be needed to clarify the kinetic basis of the altered inactive‐state behavior. It should be noted that the βY307 library was analyzed in the βT165S/G181A background, which attenuates ADP inhibition. Therefore, the inactive‐state fractions observed in this study are likely to differ from those in the wild‐type background. Nevertheless, because all βY307 variants were compared in the same background, the observed correlation between side‐chain size and inactive‐state fraction suggests that substitutions at βY307 can modulate inactive‐state behavior even under conditions where ADP inhibition is partially suppressed.

Comparison of the two saturation mutagenesis datasets highlights the distinct nature of their contributions to catalysis. For βE190, a catalytic residue directly involved in hydrolysis, mutations produced sharply defined outcomes: only the conservative aspartate substitution retained residual activity, whereas nearly all other substitutions abolished rotation entirely. In contrast, βY307 exhibited a broader tolerance to substitution, with many variants retaining detectable activity and displaying graded effects depending on side‐chain volume and hydrophobicity. This contrast underscores the fundamental difference between residues essential for chemistry at the active site and residues that modulate the efficiency or regulation of turnover. Importantly, such global trends together with informative exceptions would have been difficult to infer from a limited set of rationally chosen substitutions. Furthermore, because our assay is based on single‐molecule rotation measurements, it allows us to distinguish between two otherwise inseparable contributors to apparent activity at the ensemble level: the rotation rate during active periods and the probability of dwelling in inactive states such as ADP inhibition. This dual readout provides a more complete mechanistic picture than ensemble assays, revealing not only how specific substitutions alter the chemical step itself but also how they influence the dynamic equilibrium between active and inactive conformations.

Taken together, these results demonstrate the power of combining high‐throughput cell‐free synthesis with single‐molecule rotation assays to dissect the functional roles of individual residues in F_1_‐ATPase. Unlike conventional ensemble approaches, this method enables rapid and parallelized evaluation of comprehensive mutant libraries, while simultaneously resolving catalytic turnover rates and regulatory state probabilities at the single‐molecule level. Such capability allows residue‐by‐residue mapping of contributions to both chemistry and regulation, providing unprecedented resolution into the design principles of rotary ATPases. At the same time, this assay does not independently quantify ATP hydrolysis events that may occur without productive rotation. Conventional biochemical ATPase measurements therefore remain complementary and important for evaluating chemo‐mechanical coupling more completely.

Future applications could extend saturation mutagenesis to additional residues, explore adaptive landscapes under different physicochemical conditions, and integrate computational modeling for predictive design. Ultimately, this approach will not only deepen our mechanistic understanding of rotary catalysis but also guide the engineering of synthetic molecular machines with tailored functions.

## 4 MATERIALS AND METHODS

### 4.1 Plasmid construction

Genes encoding the α, β, and γ subunits of thermophilic *Bacillus* PS3 F_1_‐ATPase were cloned into separate pRSET‐B plasmids under the control of a T7 promoter. To enable site‐specific biotinylation, Avi‐tag sequences (GLNDIFEAQKIEWHE) were genetically introduced into the γ subunit between A108 and S109, and between G163 and T164. Mutations of βE190, βY307, and βT165S/G181A (Muneyuki et al., 2007; Nishizaka et al., 2004) were generated by custom gene synthesis (GenScript Japan) or site‐directed mutagenesis, and confirmed by sequencing. For *E. coli*‐expressed F_1_, the Avi‐tagged F_1_ expression plasmid was constructed based on the wild‐type TF_1_ expression plasmid (Tanigawara et al., 2012; Watanabe et al., 2014).

### 4.2 Cell‐free protein synthesis (PURE system)

Cell‐free transcription–translation was carried out using the PUREfrex 2.0 system (GeneFrontier). Reactions (15 μL) contained DNA plasmids at α:β:γ DNA molar ratios of 1:1:1, 1:1:2, 1:1:4.25, or 1:1:8 for optimization of the expression condition. To assist folding, the commercially available DnaK/DnaJ/GrpE chaperone system (DnaK Mix, GeneFrontier) or the GroEL/GroES chaperone system (GroE Mix, GeneFrontier) was added at 1:20 (v/v) relative to the final reaction volume. Reactions were incubated at 37°C for 3 h. For enzymatic biotin labeling, BirA ligase (300 nM) and biotin (500 μM) were added to reaction mixtures. Specific biotinylation of the γ subunit was confirmed by Western blotting in representative reactions with streptavidin–alkaline phosphatase conjugates. Prior to single‐molecule assays, reaction mixtures were diluted 10‐fold into assay buffer (50 mM 3‐(N‐morpholino)propanesulfonic acid–KOH, pH 7.0, 50 mM KCl, 2 mM MgCl_2_) supplemented with 5 mg/mL bovine serum albumin (BSA). For single‐molecule assays including multiplexed rotation assays, reactions were prepared at an α:β:γ DNA molar ratio of 1:1:2 in the presence of DnaK Mix.

### 4.3 Single‐molecule rotation assay

Flow cells were prepared using Ni‐NTA‐coated coverslips (Watanabe et al., 2023). The diluted reaction mixture after PURE expression and biotinylation or purified F_1_ solution was infused into the flow cell, where F_1_ was immobilized on the glass surface via 10 × His and 6 × His tags at the N termini of the β and α subunits, respectively. Proteins from the PURE system itself do not carry the His‐tags and were removed by washing the chamber with 60 μL of assay buffer supplemented with 5 mg/mL BSA after the 5‐min immobilization step. A 30 μL aliquot of streptavidin‐coated gold nanoparticle solution (40 nm diameter) (Watanabe et al., 2023) in assay buffer supplemented with 5 mg/mL BSA was then introduced as rotation probes through the biotin–streptavidin linkage to the γ subunit. After allowing 10 min for probe binding, rotations were initiated by infusing 60 μL of ATP solution into the flow cell and observed using a custom‐built laser dark‐field microscope with a 60× objective lens for gold nanoparticles (Ueno et al., 2010). The ATP solution consisted of assay buffer supplemented with 2 mM Mg‐ATP and an ATP‐regenerating system (2 mM phosphoenolpyruvate and 100 μg/mL pyruvate kinase). Scattered light from the gold nanoparticles was collected using a CMOS camera (FASTCAM 1024PCI, Photron) at a frame rate of 10,000 fps. Measurements were performed at room temperature (23 ± 2°C).

### 4.4 Data analysis

Images were analyzed with custom‐made software (Watanabe et al., 2023). Probe positions were determined by calculating the centroid of the nanoparticle image. Angular displacements were calculated from *x*–*y* trajectories, and rotation rates were determined as the maximum slope of the angle‐time trace obtained by linear regression within a 0.4‐s sliding window. Pauses longer than 0.1 s were defined as inhibitory pauses. The active rotation time was calculated from the durations of rotating intervals between inhibitory pauses. The fraction of time spent in the inactive state was calculated relative to the total recording time (= 20 s). Non‐rotating particles were not included in the inactive‐state analysis. Because nonspecific attachment of probe particles contributed substantially to the total observed particle count in the present assay, the ratio of rotating particles to total observed particles was not used as a quantitative estimate of the fraction of ADP‐inhibited molecules.

### 4.5 Multiplexed rotation assay and crosstalk evaluation

To test multiple variants in parallel, 1 μL aliquots of diluted PURE reactions were spotted on a Ni‐NTA–coated coverslip at 9‐mm intervals. Spots (3–4 mm in diameter) were sealed to form a flow channel with a second coverslip, using double‐sided tape as a spacer. After immobilization of each F_1_ variant in each spot on the glass surface for 7 min, the flow channel was washed by perfusion with 180 μL of assay buffer supplemented with 5 mg/mL BSA and incubated for 5 min to block nonspecific adsorption of gold nanoparticles. Then, 90 μL of gold nanoparticle solution in assay buffer supplemented with 5 mg/mL BSA was introduced and incubated for 10 min to allow binding to F_1_. Unbound gold nanoparticles were removed by perfusing the flow channel with 180 μL of assay buffer supplemented with 2 mM Mg‐ATP and an ATP‐regenerating system, after which rotation measurements were initiated. Rotation was recorded simultaneously at multiple spots using a CMOS camera (FASTCAM 1024PCI or FASTCAM NOVA S16, Photron) at a frame rate of 1,000 fps. Crosstalk was evaluated using alternating spots of F_1_(βT165S/G181A) and F_1_(βE190Q), with 10 replicates each. Crosstalk was defined as rotation events detected at F_1_(βE190Q) spots.

## AUTHOR CONTRIBUTIONS

**Mai Taguchi:** Investigation; data curation; formal analysis; visualization; writing – review and editing. **Tatsuya Oya:** Investigation; visualization; writing – review and editing; formal analysis; data curation. **Hiroshi Ueno:** Conceptualization; methodology; investigation; visualization; writing – review and editing; funding acquisition. **Hiroyuki Noji:** Conceptualization; supervision; project administration; funding acquisition; writing – original draft; writing – review and editing.

## CONFLICT OF INTEREST STATEMENT

The authors declare no conflict of interest.

## Supporting information


**Data S1.** Supporting Information.

## Data Availability

The data that support the findings of this study are available from the corresponding author upon reasonable request.
