# Supplementary material for: High‐throughput mutational analysis of F1 ‐ATPase by integrated cell‐free protein synthesis and single‐molecule rotation assay
Source: Protein Sci. 2026 Jul 10;35(8):e70699. doi: 10.1002/pro.70699 (PMC13351929; doi:10.1002/pro.70699)
Supplement: Supplementary file 1 — Data S1. Supporting Information. [file PRO-35-e70699-s001.docx]

**Supporting Information**

**High-throughput mutational analysis of F_1_-ATPase by integrated cell-free protein synthesis and single-molecule rotation assay**

Mai Taguchi^1#^, Tatsuya Oya^1#^, Hiroshi Ueno^1^*, Hiroyuki Noji^1,2^*

^1^Department of Applied Chemistry, Graduate School of Engineering, The University of Tokyo, Tokyo, Japan

^2^Research Institute of Planetary Health (RIPH), The University of Tokyo, Tokyo, Japan.

*Corresponding authors: hueno@g.ecc.u-tokyo.ac.jp and hnoji@g.ecc.u-tokyo.ac.jp

^#^ These authors contributed equally to this work.


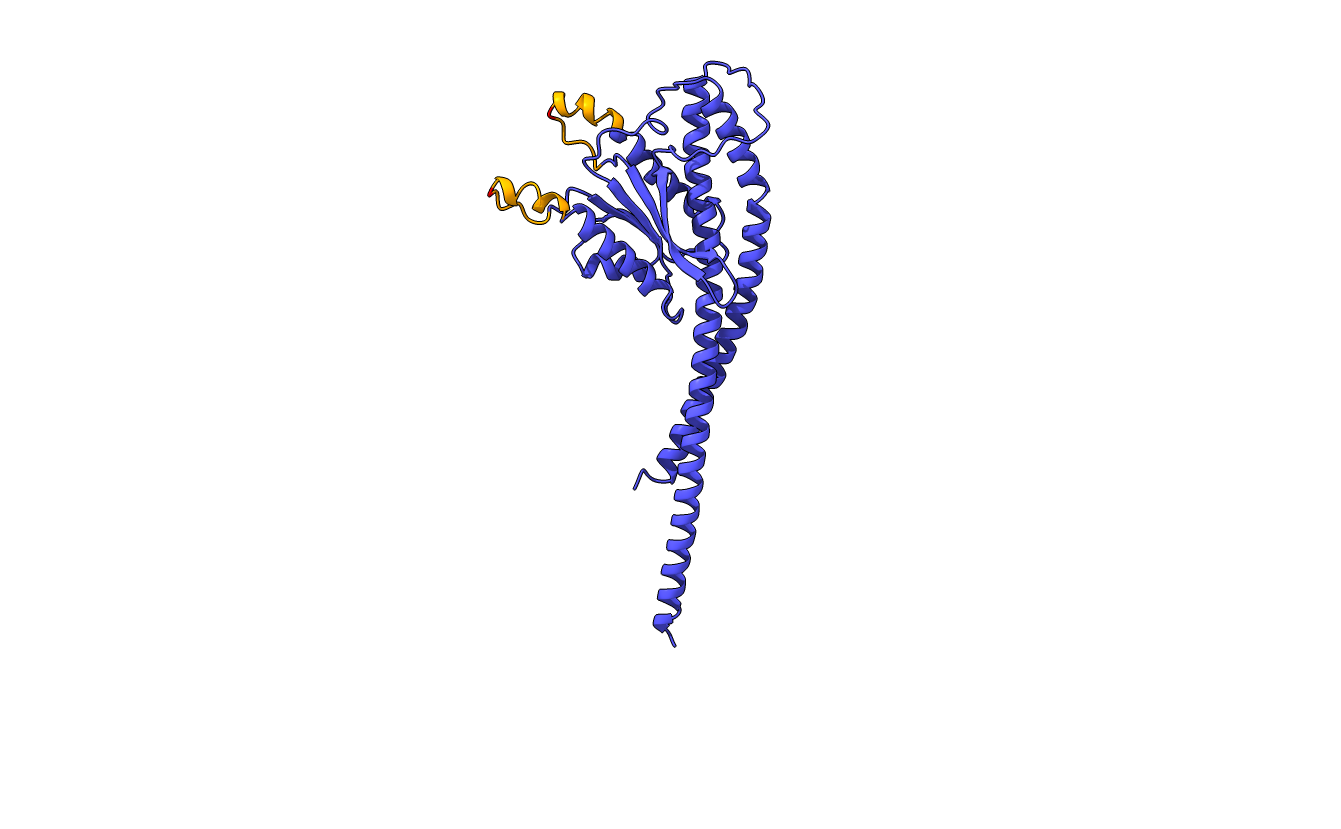


**Figure S1. AlphaFold3-predicted structure of the Avi-tagged γ subunit.**

AlphaFold3-predicted model of the Avi-tagged γ subunit showing the positions of the two inserted Avi-tag sequences. The Avi-tag regions are highlighted in yellow, and the lysine residues within the Avi-tags that are expected to be biotinylated are shown in red.


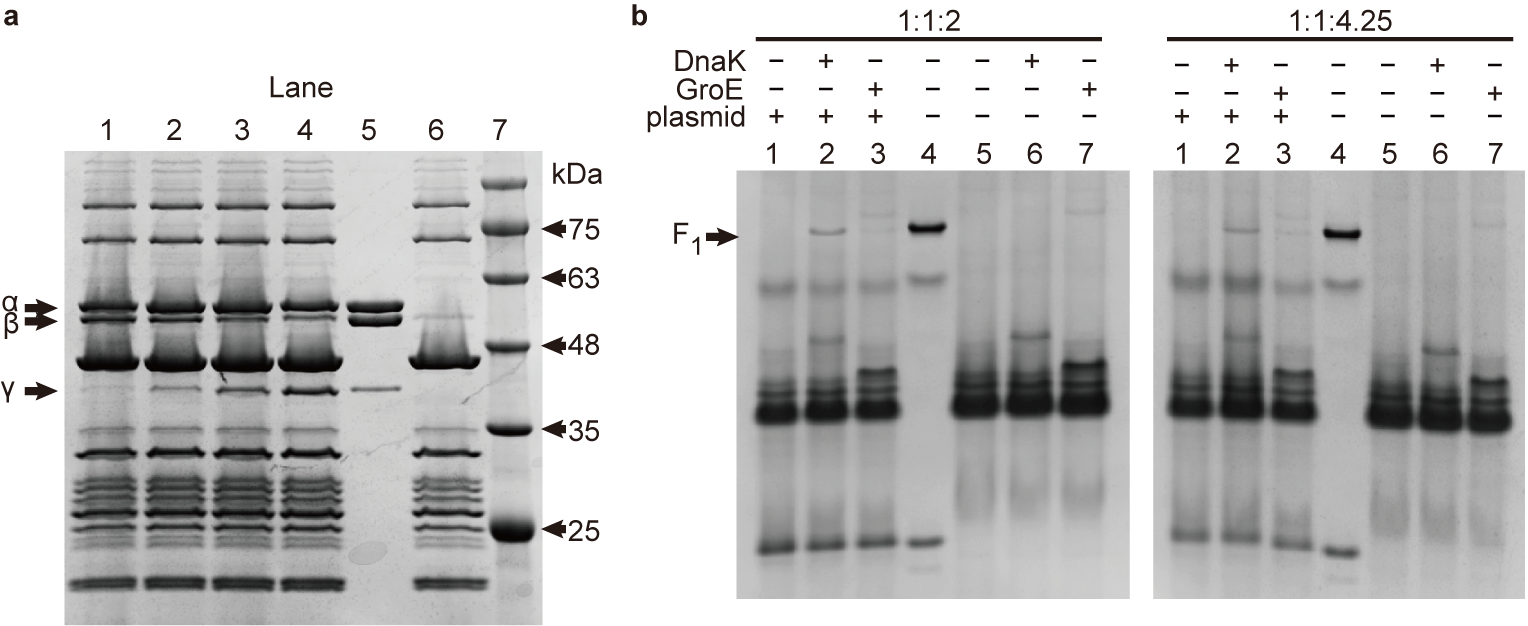
**Figure S2. Independent replicate gel analyses corresponding to Figure 1b and c.**

(a) Independent replicate of the SDS-PAGE analysis shown in Figure 1b, performed in a separate IVTT reaction on a different day. Lanes 1–4: reactions with expression vectors for the α, β, and γ subunits mixed at α:β:γ DNA molar ratios of 1:1:1, 1:1:2, 1:1:4.25, and 1:1:8, respectively; lane 5, purified F_1_; lane 6, PURE system only; lane 7, molecular-weight marker. (b) Independent replicate of the native-PAGE analysis shown in Figure 1c, performed in a separate IVTT reaction on a different day. Reactions containing the α, β, and γ expression plasmids at α:β:γ DNA molar ratios of 1:1:2 (left) and 1:1:4.25 (right) were analyzed. In each panel, lane 1, plasmids only (no added chaperone); lane 2, plasmids + DnaK Mix; lane 3, plasmids + GroE Mix; lane 4, purified F_1_; lane 5, PURE system only (no plasmids, no added chaperone); lane 6, PURE system only + DnaK Mix; lane 7, PURE system only + GroE Mix.


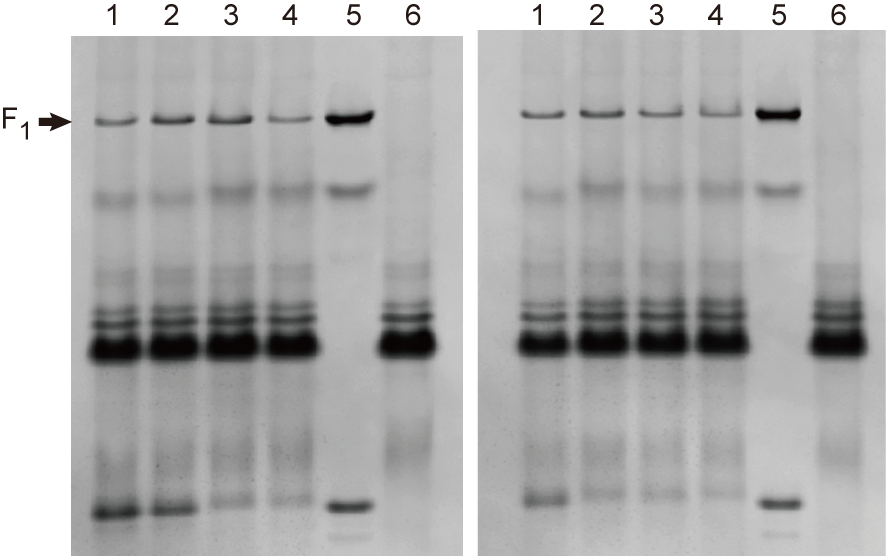
**Figure S3. Cell-free expression of F_1_-ATPase without Avi-tag in the absence of added chaperones.** Native PAGE analyses from two independent IVTT reactions performed on different days are shown (left, experiment 1; right, experiment 2). In each panel, lanes 1–4: reactions with expression vectors for the α, β, and γ subunits (without Avi-tag) mixed at α:β:γ DNA molar ratios of 1:1:1, 1:1:2, 1:1:4.25, and 1:1:8, respectively; lane 5, purified F_1_; lane 6, PURE system only. A band corresponding to the F_1_ complex was observed under all tested DNA mixing conditions in both experiments.


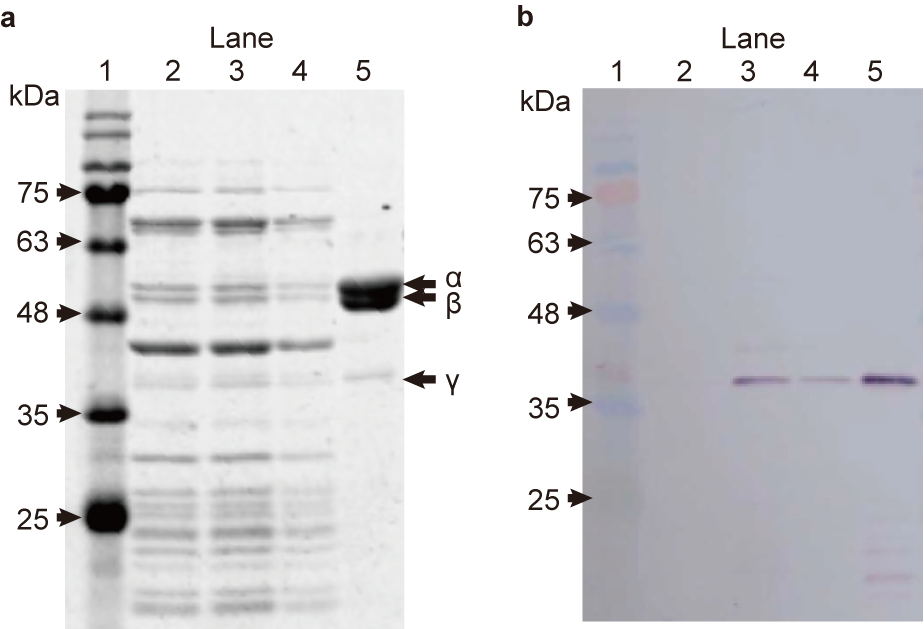
**Figure S4. Western blotting of biotinylated cell-free expressed F_1_.**

(a) SDS-PAGE of cell‑free expression products (α: β: γ = 1: 1: 4.25) followed by BirA treatment. Reactions were performed as indicated. Lane 1: molecular‑weight marker; lane 2: without BirA; lane 3: with BirA; lane 4: with BirA (2.5 times dilution of lane 3); lane 5: purified F_1_. (b) Immunoblots stained by streptavidin-alkaline phosphatase conjugates. Lane 1: molecular‑weight marker; lanes 2-4: same reactions as in (a); lane 5: biotinylated purified F_1_. Specific biotinylation of the γ subunit was confirmed.


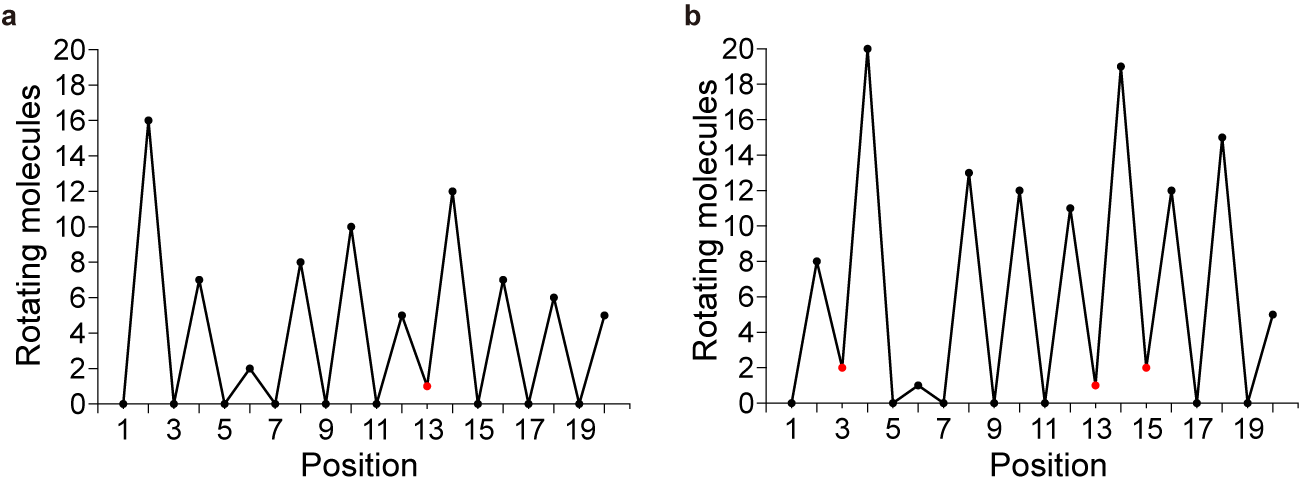


**Figure S5. Additional independent crosstalk evaluation experiments corresponding to Figure 2b.**

(a, b) Results of two additional independent crosstalk evaluation experiments performed with two independent PURE synthesis experiments. In each experiment, PURE reactions expressing F_1_(βE190Q) and F_1_(βT165S/G181A) were alternately spotted within a common flow channel (see Figure 2a). Positions 1, 3, 5, 7, 9, 11, 13, 15, 17, and 19 contained F_1_(βE190Q), whereas positions 2, 4, 6, 8, 10, 12, 14, 16, 18, and 20 contained F1(βT165S/G181A). Red marks indicate rare rotation events detected at F_1_(βE190Q) spots.


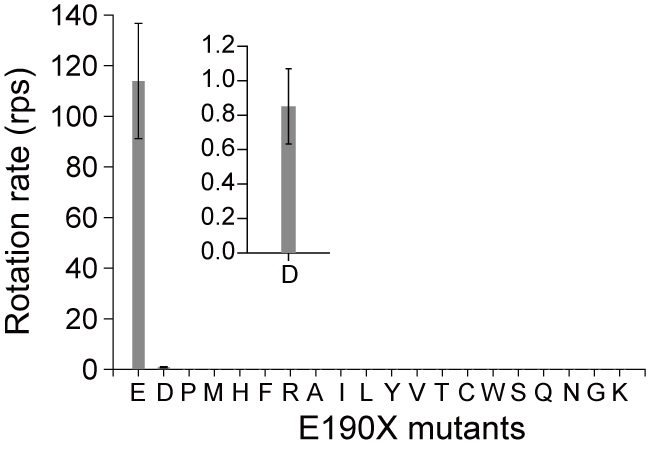
**Figure S6. Multiplexed rotation assay of βE190 site-saturation mutants.**
Rotation rates of βE190 site-saturation mutants measured by the multiplexed single-molecule rotation assay. Data from two independent datasets were combined for analysis. Each dataset was generated from an independent PURE synthesis experiment and subsequent multiplexed rotation assays. Detectable rotation was observed only for wild-type βE190 (*N* = 5) and the βE190D mutant (*N* = 15). No detectable rotation was observed for the other substitutions. Bars represent mean rotation rates and error bars indicate SD.

**Table S1. Pause statistics and active rotation times for βY307 mutants in the multiplexed single-molecule rotation assay.**

| Y307X variant | *N* (molecules) | Mean pause duration (s) | Mean active rotation time (s) | Mean number of pauses per molecule |
| --- | --- | --- | --- | --- |
| Y | 30 | 1.23 | 5.41 | 2.5 |
| K | 32 | 0.66 | 0.78 | 13.8 |
| R | 6 | 0.60 | 1.11 | 11.3 |
| H | 4 | 3.38 | 2.51 | 3.5 |
| D | 2 | 0.47 | 0.18 | 30.5 |
| E | — | — | — | — |
| S | 2 | 1.01 | 0.10 | 18.0 |
| T | 7 | 4.14 | 1.18 | 4.6 |
| C | 8 | 1.62 | 1.79 | 5.9 |
| N | — | — | — | — |
| Q | 37 | 2.57 | 2.57 | 4.0 |
| A | — | — | — | — |
| V | 6 | 2.61 | 0.13 | 7.3 |
| I | 18 | 1.22 | 0.24 | 13.8 |
| L | 25 | 1.59 | 3.29 | 3.9 |
| M | 6 | 3.09 | 1.80 | 4.3 |
| F | 11 | 2.40 | 1.76 | 4.8 |
| W | 9 | 0.39 | 2.35 | 7.0 |
| P | — | — | — | — |
| G | 8 | 2.28 | 0.16 | 8.3 |

***Pause statistics and active rotation times were calculated from 20 s of observation time for each molecule under the standard assay conditions.**
